# Anesthesia and the Developing Brain: Relevance to the Pediatric Cardiac Surgery

**DOI:** 10.3390/brainsci4020295

**Published:** 2014-04-16

**Authors:** Lisa Wise-Faberowski, Zoel A. Quinonez, Gregory B. Hammer

**Affiliations:** Lucile Packard Children’s Hospital, Stanford University School of Medicine, Palo Alto, CA 94305, USA; E-Mails: zoelq@stanford.edu (Z.A.Q.); ham@stanford.edu (G.B.H.)

**Keywords:** neurotoxicity, seizures, isoflurane, sevoflurane, ketamine, propofol, etomidate, desflurane, dexmedetomidine

## Abstract

Anesthetic neurotoxicity has been a hot topic in anesthesia for the past decade. It is of special interest to pediatric anesthesiologists. A subgroup of children potentially at greater risk for anesthetic neurotoxicity, based on a prolonged anesthetic exposure early in development, are those children receiving anesthesia for surgical repair of congenital heart disease. These children have a known risk of neurologic deficit after cardiopulmonary bypass for surgical repair of congenital heart disease. Yet, the type of anesthesia used has not been considered as a potential etiology for their neurologic deficits. These children not only receive prolonged anesthetic exposure during surgical repair, but also receive repeated anesthetic exposures during a critical period of brain development. Their propensity to abnormal brain development, as a result of congenital heart disease, may modify their risk of anesthetic neurotoxicity. This review article provides an overview of anesthetic neurotoxicity from the perspective of a pediatric cardiac anesthesiologist and provides insight into basic science and clinical investigations as it relates to this unique group of children who have been studied over several decades for their risk of neurologic injury.

## 1. Introduction

The practice of pediatric anesthesia has advanced within the last two decades [1,2,3]. The emergence of pediatric-trained individuals, with subspecialty training in pediatric critical care, advanced the specialty in the 1980s and 1990s. Subspecialty training and board-certification in pediatric anesthesia has provided pediatric anesthesia specialists, which has further validated the specialized, clinical science of providing anesthetic care to newborn infants and young children [4]. Indeed, clinical anesthetic care in these children has improved and the practice of providing anesthesia has changed, numerous anesthetics have been performed in young infants and children without obvious neurologic complications. However, beyond the clinical science, basic science may offer a different perspective [5,6,7,8].

Neurologic outcome studies in the clinical neonatal and pediatric populations have existed for several decades. These neurologic outcome studies are specific to the preterm [9,10,11] and congenital heart disease populations [12,13,14,15,16,17]. With the recent concern of anesthetic neurotoxicity in the developing brain, mostly from laboratory investigations [5,6,7,8,18,19,20,21,22] the neurologic assessment of children after anesthetic exposure at a young age is now being emphasized [23,24,25,26,27,28,29,30].

This review article provides a background to the emergence of pediatric anesthesia as a true subspecialty of anesthesia, with board certification recently available to those who have completed fellowship training in the specialty [4]. The evolution of our clinical pediatric anesthesia practice and further sub-specialization in areas such as pediatric cardiac anesthesia has led us to concerns of anesthetic neurotoxicity in those infants and children that we provide anesthesia for on a routine basis.

## 2. History

In the current era, a surgical procedure in a young infant or child performed in the absence of anesthesia would seem unethical. However, prior to the late 1980s and early 1990s minimalizing anesthetic exposure in infants and young children, due to the risk of potentially causing death as either the result of hypotension, arrhythmia, hypoxia or apnea, was not an uncommon practice [1,3,31,32,33]. It was during the late 1980s to early 1990s that the routine use of pulse oximetry became available and the sub-specialization of true pediatric anesthesiologists was emerging [1,2,34,35]. Individuals previously trained in pediatrics were now finding a career in anesthesia. This new group of pediatric-trained anesthesiologists also sought training in critical care. The rise of these triple-trained anesthesiologists, routine use of pulse oximetry and newer anesthetic agents such as rocuronium, desflurane and sevoflurane, changed the practice of providing anesthesia for children for surgical procedures [34,35,36,37]. Indeed, as the practice of pediatric anesthesia evolved, with newer agents such as sevoflurane, as opposed halothane, the anxiety level of anesthesia providers during critical periods of anesthesia, such as the anesthetic induction of an infant or young child, was reduced [3,37,38].

We had learned even with the use of halothane that an induction could go smoother with the administration of nitrous oxide [3]. Indeed, with gradual titration of an anesthetic that was less irritating to the peripheral airway, such as halothane, and, in present day, sevoflurane, anesthesia could be more safely provided to even extremely premature neonates [37]. Moreover, as we gained comfort with anesthetic induction in these young infants and children, we began to add “cures” for preoperative anxiety, which included the use of a premedication with midazolam or the parental presence in the operating room during anesthetic induction [36,39,40]. Indeed our practice had evolved to such that with shorter recovery times and improved analgesia care, children could be discharged home the same day after surgery [1,40].

However, a new phenomenon began to emerge and that was the presence of emergence delirium [41,42,43,44,45]. Our panacea for a smoother induction, shorter anesthetic course was now troubled with a postoperative concern. The etiology for this phenomenon was uncertain, but it was felt to be related to the rapid emergence from sevoflurane [43,44,45]. The idea of the young brain being disrupted or “scrambled” by an anesthetic exposure was a new concept. However, the concern of an adverse reaction on the developing brain of a young infant or child became an ever-present concern.

In early 2003, a landmark article by Jevtovic-Tedorovic, stated that the “triple-cocktail of anesthetics” *i.e.*, midazolam, nitrous oxide and isoflurane, promoted neuronal cell death and long-term memory deficits in a neonatal rat pup model of developing brain [5]. What followed from this was a flurry of *in vitro* and *in vivo* studies further confirming that isoflurane, when given for 5–6 h, at a critical period in development, 4–6 days in a rodent, caused anesthetic neurotoxicity [7,18,22]. These manuscripts were criticized mainly for their inability to mimic the “true “conditions of an anesthetic in an infant or young child. Then, a series of retrospective investigations in children emerged that supported or refuted the possibility that anesthetic exposure at an early age in development may be harmful [23,25,26,27,28,30]. Retrospective analyses are flawed by their inability to truly define a causal relationship between the exposure, in this instance anesthesia, and the outcome, neurodegeneration. A recent editorial, in Anesthesia and Analgesia, further noted the difficulties with retrospective investigations, evaluating anesthetic neurotoxicity in young infants and children, and emphasized the need for prospective-randomized trials to more accurately address our concerns [46].

## 3. Prospective-Randomized Trials

Prospective-randomized trials assessing neurologic outcome in infants and children is not new to the specialty of pediatrics. The longest, prospective, neurologic follow-up of infants after surgery and anesthesia is that of the Boston Circulatory arrest trials [12,13,14,15]. There are a number of confounding variables and the impact of anesthesia specifically was not addressed; however, these studies do provide some insight to our current dilemma, “Does anesthetic neurotoxicity exist in humans?” Prior to the initiation of this trial, some of the same investigators evaluated the stress response in neonates undergoing surgical repair of congenital heart disease [47,48]. The stress response in these infants was extremely high and was felt to be associated with increased morbidity, later psychosocial abnormalities and mortality [47,48,49,50,51,52]. This prior investigation, evaluating the stress response in infants, prompted the use of deeper anesthesia in these children.

## 4. Pain and the Stress Response

The 1990s saw a departure from the practice of surgery without anesthesia. The notion that neonates could not perceive pain was no longer withheld [1,31,47,48]. It became apparent that repeated pain and stress in neonates promoted excessive NMDA excitation, resulting in damage to developing neurons [47,48]. It was further proposed that repeated stress and pain in a neonate would result in later behavioral difficulties [52,53]. These neonates later suffered from impaired social skills, attention deficit hyperactivity disorder and self-destructive behavior [12,50,52,53]. Indeed, in later follow-up, the infants presumed at risk of poor neurologic outcome, in the Boston Circulatory Arrest Trial, suffered from an inability to incorporate themselves within society. Though a retrospective evaluation, the conclusion is that anesthesia exposure in early life, may lead to hyperactivity/attention deficit disorder during adolescence and adulthood [26,30,46,54]. This begs the question: was it the pain and stress or its treatment that resulted in later observations of psychosocial abnormalities in these children [54,55]?

The Boston Circulatory Arrest Trial was performed in the era of using high dose opioids for surgical repair of congenital heart repair requiring cardiopulmonary bypass [17,47]. Though the minimalist approach to providing anesthesia to children was now being abandoned in the 1990s, comparisons between approaches to anesthesia offering the best neuroprotection were not being investigated until early 2000 [56,57,58]. The importance of the type of anesthetic used during cardiopulmonary bypass and neurologic outcome was identified by Kurth *et al.* and Loepke *et al.* [57,58]. In a piglet model of deep hypothermic circulatory arrest with low-flow cardiopulmonary bypass, two anesthetic strategies were compared: fentanyl-droperidol (F/D) and desflurane [58]. Desflurane conferred neuroprotection in a neonatal piglet model of cardiopulmonary bypass. Since these investigations, comparisons of anesthetic techniques in assessing neurologic outcome in neonatal animal models of cardiopulmonary bypass have not been addressed.

In 2003 the idea of anesthetics as potential “neuroprotectants” of the developing brain changed [5]. Pediatric cardiac anesthesiologists were now confronted with a new and different issue related to prolonged and repeated exposure to anesthetic agents in infants and young children who are at an age of critical brain development.

## 5. Electroencephalogram

What can we learn from past experience? Reflecting back to the Boston Circulatory arrest trial, there was a group of infants noted to be at risk for poor neurologic outcome. These infants were defined by the presence of subclinical seizure activity in the post-operative period [17]. Still, little is known about the interpretation of EEG in children undergoing anesthesia. In a later series of infants and children, EEG analysis was obtained during isoflurane and sevoflurane anesthesia [59]. It was noted that the emergence patterns, both global and regional differed in these two anesthetic groups. Not only were there anesthetic specific differences in alpha, delta and theta power, but concentration differences were seen in the alpha and delta EEG patterns. This study did not have enough power to determine age-dependent effects; but the concentration effect in this investigation cannot be overlooked as potentially insinuating a dose-dependent effect that may impact neurocognitive outcomes [59].

## 6. Ventilation and Surgery

Another reason to mention these studies, in which children were stressed either from mechanical ventilation (neonates) or surgery (congenital heart population), was the concern with Jevtovic-Tedorovic study that the animals were not mechanically ventilated and lacked a surgical stimulus [5,36]. Several animal investigations (*in vitro* and *in vivo*) followed to assess independently the effect of pH and carbon dioxide control, and surgical stimulation and its effect on anesthetic neurotoxicity [18,22,60,61,62,63,64]. These studies found that in the presence of pH control, isoflurane had an age-and duration-dependent effect on anesthetic neurotoxicity [18,22]. Hypercarbia, alone, promoted neurodegeneration similar to that observed with anesthesia alone in PND7 rat pups [62,63]. The use of carbon dioxide on cardiopulmonary bypass machines to enhance cerebral blood flow, may have its own effect on brain development [64,65]. Finally, anesthetic-induced neurodegeneration can be reduced or exacerbated by a surgical stimulus [60,61].

## 7. The Choice of Anesthetic

The most widely studied are volatile anesthetics, especially isoflurane, in terms of anesthetic neurotoxicity [66,67,68,69,70]. These anesthetics have been implicated because of their combined activity as both an NMDA antagonist and GABA agonist. Investigations with isoflurane have included rodents and more recently non-human primates [71,72,73]. Evidence is conclusive in developing models of *in vitro* and *in vivo* rodent brain that isoflurane provides a duration- and dose-dependent effect on neuronal cell death [5,18,22]. Studies have looked at sevoflurane, independently and in comparison to isoflurane, and have found neuronal cell death as well [6,8,19,20,21,66,68,70]. Long-term outcome appears to be potentially similar with both agents but early or short-term memory may not be impaired with sevoflurane [21]. Similarly, an equipotent dose of desflurane was evaluated and found to be equivalent to isoflurane and sevoflurane in caspase-3 mediated cell death [68]. However, long-term behavioral and short-term memory impairment has not been demonstrated with desflurane [71].

Isoflurane has been the most studied of the volatile anesthetics [5,22,66,71,72]. Regional distribution of cell death, as the result of isoflurane exposure, in the developing brain has been observed, with continued effects that exist into young adulthood, in areas of the dentate gyrus and olfactory bulb, which continue to be sites of neurogenesis throughout development [67]. This information increases the concern that the window of vulnerability of the areas of brain at risk for anesthetic neurotoxicity may be greater than that previously realized. However, even in these animal models of anesthetic neurotoxicity, the potential for neuronal cell recovery exists [66,73]. It appears that environment may be a key contributor to the deficits observed in these animals [74]. As has also been seen in the adult literature the potential for preconditioning also exists [66]. In rodents, preconditioning with xenon or even isoflurane can ameliorate these deficits [66].

In nonhuman primate models, isoflurane has shown evidence of not only neuronal degeneration but involvement of oligodendrocytes as well [71,73]. Structural involvement includes the cerebellum, caudate putamen, amygdala and cerebrocortical regions as well. The white matter involvement with exposure to isoflurane was observed not only in neonatal but also fetal rhesus macaque brain as well [71,72,73]. The nonhuman primate model has been the first to provide insight into the potential for difficulties in myelination and white matter injury as a result of anesthetic exposure at a young age in brain development. Interestingly to note, is that the oligodendrocyte injury in the fetal brain was more diffuse than that observed in the neonatal brain [71,73]. This is of particular concern to pediatric cardiac anesthesiologists. There is continued evidence that the developing brain of infants with congenital heart disease parallels that of the premature infant [64,74,75,76,77]. White matter injury prior to cardiopulmonary bypass and anesthetic exposure is further exacerbated postoperatively [78,79].

## 8. Opioids

When comparing desflurane to opioid-based anesthetic techniques in a piglet cardiopulmonary bypass model, desflurane conferred neuroprotection [57,58]. This model differs in not only the species of animal used but also the stressor (cardiopulmonary bypass) used during anesthetic exposure. There have been no recent investigations comparing anesthetic neurotoxicity to that induced by opioids. The effect of opioids on non-fetal brain development has not been fully evaluated [49,50]. One recent investigation showed that opioids have an effect on the developing cortex and amygdala but areas such as the hippocampus, which has importance in memory formation, is spared [80]. Though *in* *utero* opioid exposure tends to affect oligdendrocytes, the glial cells are relatively spared in the neonatal brain [81]. Long-term neurologic follow-up of neonates exposed to morphine early in development shows neither a protective or harmful effect of repeated and continuous neonatal exposure to morphine [49].

## 9. Midazolam

In the neonatal population the NOPAIN trial evaluated the effect of analgesia on neurologic outcomes [31,49,50,51]. The patients were randomized to receive morphine, midazolam or placebo. The three groups did not differ in their severity of illness score or socioeconomic status. In the initial, single institution trial, poor neurologic outcomes occurred in 24% of neonates in the placebo group, 32% in the midazolam group, and 4% in the morphine group (likelihood ratio χ^2^ = 7.04, *P* = 0.03). In a study of neurodevelopmental outcomes of infants at 12 months of age who had undergone the arterial switch operation, total midazolam dose adversely affected neurodevelopmental outcome [82,83]. However, when assessing the entire study population, the Bayley-III composite score was within the range of the normal population [82,83]. Additional follow-up of this cardiac cohort has not occurred. Midazolam use in infancy may produce a detrimental effect on the developing brain. In an earlier study in the pediatric cardiac population, the addition of midazolam to an opioid-based anesthetic did not further diminish the stress response [48]. The “midazolam effect” is mentioned because in the initial study by Jevtovic-Tedorovic, the triple cocktail (nitrous oxide, midazolam and isoflurane) promoted the greatest injury in postnatal 7 day-old rat pups when compared to isoflurane, alone [5].

## 10. Ketamine

The routine use of ketamine as a sole anesthetic agent is not common practice for many anesthesiologists. Ketamine is most frequently used as an adjunctive agent in pediatric cardiac anesthesia [84,85,86]. The purported anesthetic neurotoxicity associated with ketamine because of its NMDA antagonism has been described in several rodent models [60,70]. It has been observed that the apoptotic properties of ketamine can be attenuated with concomitant surgical stimulation [60]. Apoptosis in the fetal and neonatal rhesus macaque brain has been seen with ketamine [87]. Similar to isoflurane, the fetal brain was much more vulnerable to neurodegeneration induced by ketamine exposure. Also the pattern of neuronal loss after a 5-h exposure to ketamine was different in the two developmental ages [71,72,73]. Ketamine’s effect on white matter injury was not investigated. However, using nuclear magnetic resonance spectroscopy in children randomized to receive ketamine or not during cardiopulmonary bypass, ketamine did not confer neuroprotection nor promote neurotoxicity [24].

## 11. Propofol and Etomidate

Propofol-induced anesthetic neurotoxicity has been less well described in the rodent literature [88,89]. One study demonstrated increases in neuroapoptosis in immature mouse brain from propofol via alterations in brain-derived neurotrophic factor [90]. The rhesus macaque model has been useful in elucidating the effects of propofol on the fetal and developing neonatal brain [91]. Similar to isoflurane, the neurodegenerative effects are age-dependent with an age-specific cell death distribution pattern and oligodendrite involvement. Interestingly, when compared to isoflurane, cell death induced by 5 h of propfol administration was one-fourth that observed in the isoflurane treated nonhuman primates [92].

To date there are no studies evaluating the effect of etomidate on the developing brain. There are increasing concerns that stress and the release of cortisol could contribute to the observed effects in cell death associated with anesthetics [92]. Others hypothesize a disruption in the adrenal axis resulting in an imbalance of mineral- and glucocorticoids Though it is known that cardiopulmonary bypass disrupts the hypothalamic pituitary axis, the use of steroids on cardiopulmonary bypass ameliorates these potential deleterious effects on the developing brain [48,93,94]. This contradiction in steroid use is present in the neonatal literature as well; however, reports of poor neurologic outcomes with the neonatal use of steroids do exist [9,10,11,52].

## 12. Dexmedetomidine

There are no studies to date that demonstrate a neurotoxic effect of dexmedetomidine [70]. In fact, appears to modulate neuroprotection [95,96]. Dexmedetomidine, unlike benzodiazepines, does not disrupt REM sleep and is associated with neuroprotection in animal models of anesthetic neurotoxicity [97].

## 13. Seizures and EEG

In the Boston Circulatory Arrest Trials postoperative seizure activity, especially subclinical seizure activity, was pathognomic of poor neurodevelopmental outcome in children after anesthesia and surgery for congenital heart disease [12,13,17]. Interestingly, in a follow-up study evaluating seizures in children with congenital heart disease, no evidence of seizure activity was observed by the authors [48]. The authors attributed this to the concurrent use of midazolam in the intensive care unit. The investigators at the University of Florida, led by Dr. Anatoly Martynyuk, have been most influential in their novel developmental model of rodent EEG and its use in assessing anesthetic neurotoxicity. The authors have observed spontaneous seizure activity in neonatal animals exposed to isoflurane and sevoflurane [21]. Seizure activity with sevoflurane exposure was greater than that observed in neonatal rodents exposed to isoflurane. These authors have also shown a linkage to the hypothalamic adrenal axis with sevoflurane induced seizure activity and cell death [92]. In particular, increased aldosterone production with sevoflurane or exogenous administration of aldosterone promoted seizure activity. Seizure activity induced by sevoflurane could be reduced with the administration of bumetanide or oxytocin [21,91,98]. However, in isoflurane treated rats bumetanide had no effect on seizure activity but reduced the number of apoptotic cells [21].

## 14. Hypothermia

Hypothermia is already vital in the prevention of ischemic/reperfusion injury in neonates, infants and children undergoing repair of complex cardiac lesions using CPB or circulatory arrest. It has been shown that significant hypothermia can prevent anesthesia-induced neurodegeneration in rodents [99] and that it may be neuroprotective even when applied shortly after the insult. Thus, our current practices may already protect against anesthesia-induced neuronal apoptosis in the developing brain; and the question is likely more relevant to children with or without congenital cardiac lesions undergoing non-cardiac surgery. Nevertheless, this concept has never been studied in humans. But it follows that if found to be effective at preventing anesthesia-induced neuronal cell death, we would need to define the timing and degree of cooling, and whether active or simply passive cooling, are necessary to gain the potential benefit. This, of course, would be weighed against the potential consequences of hypothermia, such as bleeding or even the inhibition of the programmed cell death naturally occurring in the developing brain.

## 15. Investigations in Infants and Children

At present, we have only observational analyses and retrospective investigations evaluating the effects of anesthetics and neurodevelopmental outcome [16,23,25,26,27,28,29,30,54]. The initial intent of the databases used for these investigations was not to look at neurodevelopmental development in children after an anesthetic exposure. Anesthetic exposures are difficult to enumerate in terms of duration, anesthetics used and number of anesthetic exposures. Our anesthetic practice has changed and the triple cocktail of the Jevtovic-Tedorovic investigation may not be used by many providing anesthetic care to children, today [5]. Anesthesia databases represent only certain subsets of these populations and may be biased based on factors even such as gender [46]. Indications and durations of surgery in addition to other perioperative factors could not be controlled as would be if performed in a prospective fashion.

The retrospective investigations do have three prevailing themes: attention deficit hyperactivity disorder, dose- and age-dependency, and disabilities in language acquisition and abstract reasoning. A retrospective study in infants who underwent complex cardiac surgery published by Guerra *et al.* attempted to look at the relationship between total anesthetic or sedative dose and ABAS-GAC scores, or being “significantly delayed” either on the Bayley-2nd Edition (BSID-II) or Bayley-III, and found no relationship [100]. The authors cite as a limitation, however, the change from BSID-II to Bayley-III during their study, which prevented them from using the Bayley composite scoring as a continuous variable. A recent follow up investigation by Guerra *et al.* did find an association between days on chloral hydrate and performance intelligence quotient, as well as between total benzodiazepine dose and verbal motor integration scores, on 54-month follow-up of children who underwent complex cardiac surgery during infancy [101]. Bong *et al.* recently published an evaluation of Singaporean children who were exposed to anesthesia in infancy, and whose academic performances were evaluated at the age of 12 [26]. There was no difference between anesthetic exposed and non-exposed individuals on an academic achievement examination; however, exposed individuals had a four-fold greater likelihood to be diagnosed with a learning disability. This investigation prompted an editorial [46] highlighting the inability to accurately define and hence to diagnose what is considered a learning disability. The term attention hyperactivity disorder for many is a catch-all term applied to those individuals who do not perform well in school. The retrospective evaluation by Andropoulus *et al.* is the most recent investigation to be published and looks at a cohort of 59 neonates who underwent complex cardiac surgery, had preoperative MRI, a 7-day postoperative MRI and 12-month neurodevelopmental testing with Bayley-III composite scoring [102]. Their finding that volatile anesthetic exposure was associated with lower cognitive scores at 12 months highlights the possibility of volatile anesthetic as a modifiable factor affecting neurocognitive development, as well as the need for further research into this topic.

## 16. Conclusions

In summary, the practice of pediatric anesthesia has evolved over the last two decades into a more specialized care of infants and children. Though laboratory investigations have shown repeatedly that prolonged anesthetic exposure promotes neurodegeneration and memory deficits, the clinical manifestations of anesthetic neurotoxicity remain elusive. Prospective neurologic assessment in children exposed to anesthesia during early brain development seems imperative and lessons learned from neurologic outcome studies in neonates and children with congenital heart disease need to be incorporated in our anesthetic care of children. Special attention to those children with congenital heart disease seems prudent in addressing the issue of anesthetic-induced neurotoxicity as lessons learned from the past, may bring answers to our questions in the future.
